# Controversies in the Interpretation of Liquid Biopsy Data in Lymphoma

**DOI:** 10.1097/HS9.0000000000000727

**Published:** 2022-05-13

**Authors:** Maria Cristina Pirosa, Sven Borchmann, Fabrice Jardin, Gianluca Gaidano, Davide Rossi

**Affiliations:** 1 Division of Hematology, Oncology Institute of Southern Switzerland, Bellinzona, Switzerland; 2 Laboratory of Experimental Hematology, Institute of Oncology Research, Bellinzona, Switzerland; 3 Department I of Internal Medicine, Center for Integrated Oncology Aachen Bonn Cologne Duesseldorf, Medical Faculty and University Hospital Cologne, University of Cologne, Germany; 4 Cancer Center Cologne Essen – Partner Site Cologne, CIO Cologne, University of Cologne, Germany; 5 Department of Hematology, Centre Henri Becquerel, Rouen, France; 6 Division of Hematology, Department of Translational Medicine, University of Eastern Piedmont, Novara, Italy; 7 Faculty of Biomedical Sciences, Università della Svizzera italiana (USI), Lugano, Switzerland

## Abstract

The rapid evolution of genomic technologies over the last years has led to the development of different methods for the detection, measurement and analysis of cell-free DNA fragments (cfDNA) which are shed into the bloodstream by apoptotic cells and circulate at a low concentration in plasma. In cancer patients, the proportion of tumor-derived cfDNA is defined as circulating tumor DNA. This analysis, commonly known as liquid biopsy, allows to access tumor DNA through a simple blood sampling and therefore without the need of an invasive tissue biopsy. For this reason, this tool may have several clinical applications in terms of diagnosis, prognosis, and monitoring of minimal residual disease. However, there are still several critical issues that need to be resolved. In this review, we will discuss some of the controversies around this method and its potential clinical applications.

## INTRODUCTION

### What does “liquid biopsy” mean?

Liquid biopsy is a cancer test performed on a sample of blood. Historically, the blood of patients with leukemic hematological malignancies has represented a source of circulating tumor cells (CTC), thus constituting an opportunity for simple, noninvasive analysis of their genetic profile.^1,2^ In nonleukemic, solid tumors, noninvasive screening and postoperative follow-up has mainly relied on circulating protein markers, such as prostate-specific antigen,^2^ cancer antigen 15-3^3^ and others.

Along with cells and proteins, blood also contains fragments of nucleic acids that circulate free in its plasma fraction. Circulating cell-free DNA (cfDNA) was discovered in 1948 in the blood of healthy individuals,^4^ but only in 1977, it was recognized that patients with malignancy have higher levels of cfDNA compared with healthy individuals.^5^ A potential role of cfDNA in profiling a tumor genetically was first demonstrated in 1994 following the identification of tumor-associated RAS gene mutations in the plasma of patients with myelodysplastic syndrome and acute myeloid leukemia.^6,7^ Several years after, Lawrie et al^7^ showed for the first time that tumor-associated microRNAs are also detectable in the peripheral blood of lymphoma patients. These discoveries led to an increasing number of studies that, using more sensitive methods, investigated the utility of cfDNA for the characterization, monitoring and therapeutic targeting of both hematologic and solid malignancies.^8^

Accessing cancer DNA through a venipunction grants several opportunities beside its obvious convenience for both patients and physicians. Compared with the cellular fraction of blood, cfDNA is a more abundant source of tumor genetic material in cancers lacking CTC as several solid tumors and lymphomas. Compared with circulating protein biomarkers, cfDNA is more specific because it harbors the tumor fingerprint made up by its somatic mutations. Compared with single biopsies in primary cancer sites, cfDNA can be longitudinally assessed and can provide a more complete description of the cancer mutational profile; this may be of particular importance in the case of spatially and genetically heterogeneous malignant tumors. The rapid evolution of genomic technologies over the past 2 decades has led to the development of methods for the measurement and analysis of circulating tumor DNA (ctDNA) that supported a significant progress toward potential clinical applications. CtDNA analysis provides quantitative information about the tumor burden and is useful for detection of minimal residual disease (MRD) and occult metastases.^9^ Moreover, ctDNA analysis identifies mutations, amplifications, deletions and translocations, and allows the detection of genetic alterations associated with response to treatment, also known as predictive biomarkers.^10^ In this field, the milestone was represented by the Food and Drug Administration (FDA) approval in 2016 of the first blood ctDNA test based on specific epidermal growth factor receptor (*EGFR*) mutations, able to guide the use of EGFR-tyrosine kinase inhibitors in patients with non–small-cell lung cancer.^11^ More recently, other assays were approved by FDA namely companion diagnostic tests, including assays for the detection of *PIK3CA* mutations in breast cancer.^12^

While ctDNA technologies have entered in the management of a limited number of solid tumors, their analytical and clinical validation in lymphoma still remains a critical open question. In this controversy article, we aim to provide an overview of the currently debated aspects around: (i) the physiology and pathophysiology of cfDNA in lymphoma; (ii) the impact of preanalytics on ctDNA assay results in lymphomas; (iii) the technical validity, and the real-time feasibility of state-of-the-art ctDNA assays; and (iv) the clinical utility of state-of-the-art ctDNA assays to guide lymphoma diagnosis, treatment tailoring, and residual disease identification.

## WHAT IS THE PHYSIOLOGY AND PATHOPHYSIOLOGY OF CELL-FREE DNA IN LYMPHOMA?

cfDNA refers to DNA that is freely circulating in plasma. In patients with cancer, the proportion of tumor-derived cfDNA is termed ctDNA. Various forms of cell death, including apoptosis, are the predominant source of ctDNA.^13^ The amount of cfDNA in plasma corresponds to hundreds genomic equivalents per ml of plasma and is highly fragmented. The size of cfDNA varies from ~40 to 200 base pairs (bp), with a peak at approximately 166 bp. On its release, cfDNA is fragmented and nucleosomes, transcription factors, and other DNA binding proteins prevent random cleavage, resulting in specific patterns of fragmentation. These fragmentation profiles reflect chromatin proteome occupancy maps and epigenetic fingerprints. Epigenomic footprinting through both nucleosome occupancy inferred from cfDNA fragmentation patterns and methylation profiling point to the hematopoietic lineages as a major source of cfDNA in healthy subjects.^14–18^

The distinction of ctDNA from background cfDNA released by hematopoietic cells may be confounded by biological signals arising from clonal hematopoiesis. Clonal hematopoiesis is a common aging-related phenomenon where somatic mutations in hematopoietic stem cells are clonally propagated to their progeny. High-sensitivity, deep next-generation sequencing (NGS) approaches used in ctDNA mutation recovery can identify biological signals from clonal hematopoiesis in up to 90% of cancer patients and must be distinguished from tumor associated mutations. Consistently, a large fraction of cfDNA mutations recovered in cancer patients have features consistent with clonal hematopoiesis, which in turns can result in inaccurate ctDNA quantification and mutational signature recovery. Joint analysis of cfDNA and matched leukocytes distinguishes background mutations due to clonal hematopoiesis and allows accurate variant interpretation and recovery of lymphoma-associated mutations.^19^

The concentration of cfDNA in blood varies significantly. It ranges between 0 and 100 (median ~10) ng/mL in healthy subjects, and is usually elevated, though with great variability, in patients with lymphoma, ranging from 5 and 100 (median ~25) ng/mL. The fraction of lymphoma ctDNA constitutes ~0.1%–90% (median ~5%) of cfDNA. Therefore, in most lymphoma patients, a minor part of total cfDNA consists of ctDNA, though there is a great variability in the ctDNA/cfDNA ratio depending on tumor bulk and histology. Indeed, the ctDNA/cfDNA ratio is higher in patients with larger tumor volumes and/or aggressive histologies compared with patients with low tumor volume and/or indolent histologies. Given the typical low ctDNA/cfDNA ratio, highly sensitivity wet-lab approaches and bioinformatics pipelines are needed for the accurate quantification and mutation profiling of ctDNA.^20^

### Controversy

#### Impact of tumor biology on the release of ctDNA

**Figure 1.**
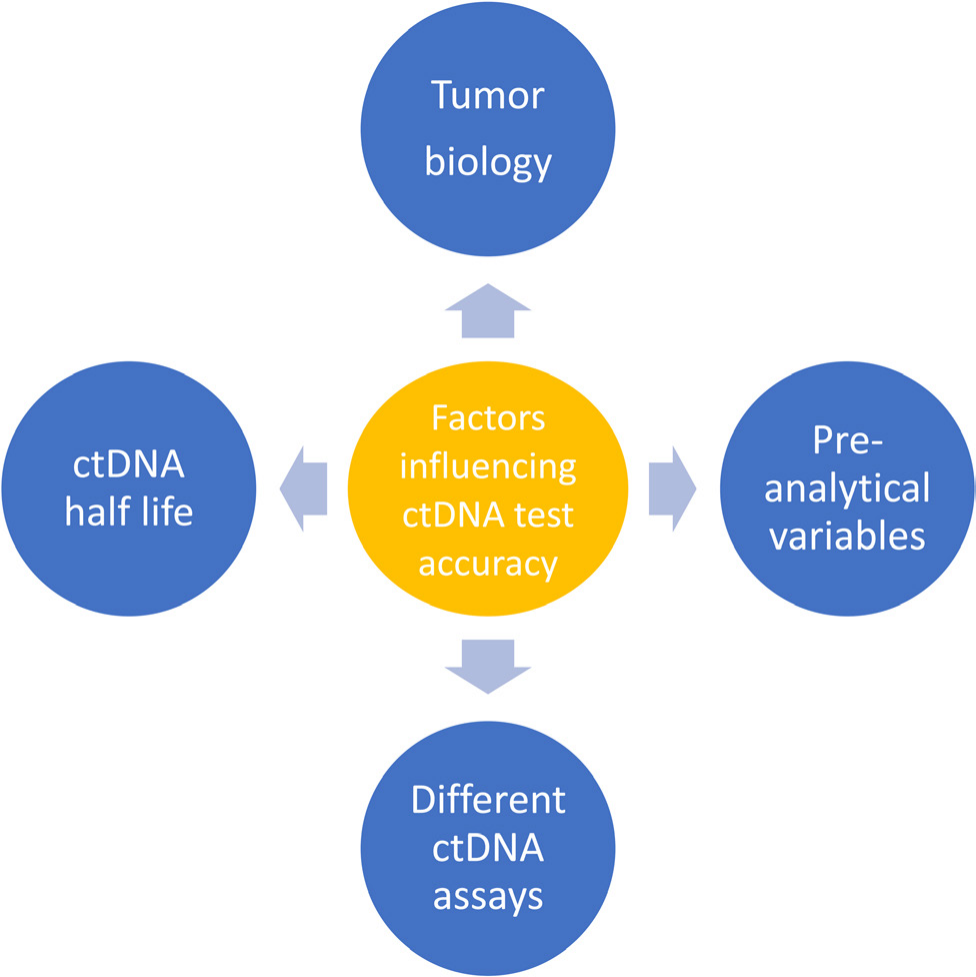
**Factors influencing the clinical applicability of liquid biopsy in lymphomas.** CtDNA = circulating tumor DNA.

The tumor type has an impact on the release of ctDNA. In solid tumors, ctDNA is detectable in >75% of patients with pancreatic, ovarian, colorectal, bladder, gastroesophageal, breast, melanoma, hepatocellular, and head and neck cancers, but in less than 50% of primary brain, renal, prostate, or thyroid cancers.^21^ Consistently, levels of ctDNA also vary according to different histological subtypes of lymphoma, with higher levels in aggressive lymphomas such as in diffuse large B-cell lymphoma (DLBCL), classical Hodgkin lymphoma (cHL), and mantle cell lymphoma (MCL) in comparison to follicular and other indolent lymphomas which have circulating DNA levels similar to healthy controls.^22–24^ Compared with solid tumors, several lymphoma types release higher amounts of ctDNA.^23,25–27^ Stage of disease and tumor burden have also an impact on cfDNA levels, which are higher in advanced stage lymphomas than in limited stage disease, and in overt progressive disease than in a disease in clinical response after the treatment.^22,28,29^ Collectively, these data indicate a relationship between tumor burden and extension and ctDNA release. Aggressive tumors with rapid turnover of proliferation and apoptosis usually have the highest levels of ctDNA (Figure 1).

The role of tumor biology and tumor–microenvironment interactions in favoring the release of ctDNA is still poorly understood in solid tumors and has never been addressed in lymphoma. In colorectal cancer (CRC), the concentration of ctDNA varies considerably and it may also depend on the ratio of tumor malignant cells to tumor microenvironment cells.^30^

#### ctDNA half-life compared with total cfDNA

A systematic analysis of the best timepoint for measuring ctDNA after tumor treatment is lacking. Release of ctDNA into the circulation depends on the location, size, and vascularity of the tumor, leading to a difference in ctDNA levels among patients. cfDNA is quickly cleared from plasma by organs, such as the liver, spleen, kidney. Therefore, cfDNA has a short and variable half-life, which ranges from 16 minutes to 2.5 hours.^31^ This effect has direct implications on the collection timing following lymphoma treatment when ctDNA is used as a surrogate biomarker of tumor reduction.

Among different studies ctDNA measurements were performed at different timepoints after treatment administration. The heterogeneity in lymphoma types included in these studies, the divergent timepoints of collection during treatment, as well as differences in preanalytics complicate the meta-interpretation of these data. In DLBCL and HL, several studies showed that a drop of less than 2-logfold in ctDNA after 1 or 2 courses of chemotherapy was associated with worse outcome.^29,32^ In one study that evaluated the efficacy of panobinostat in DLBCL, a significant increase of ctDNA on day 15 as compared with baseline was associated with lack of response to treatment.^33^ A phase I/II trial investigating anti-CD19 CAR-T cells in DLBCL showed that ctDNA levels assessed at 28 days after the product infusion were predictive of outcome.^34^ Therefore, the identification of the best timepoint to evaluate ctDNA is still a controversy, and likely depends on the type of underlying disease, type of treatment and its scheduling (Figure 1).

## HOW DO PREANALYTICS AFFECT CTDNA ASSAY RESULTS IN LYMPHOMA?

Preanalytical variables with regard to ctDNA-based assays include all steps preceding the analysis of the specimen. Preanalytical variables that reduce the ctDNA/cfDNA ratio affect the sensitivity of laboratory tests aiming at identification, quantification, and qualification of ctDNA (Figure 1). Physiologically, cfDNA levels have diurnal variations, peaking during night and increasing during exercise as well as during pregnancy. In non-neoplastic conditions, elevated cfDNA may occur during inflammation and tissue damage (ie, trauma or infraction).^35^ In lymphoma patients, such concomitant conditions may decrease ctDNA/cfDNA ratios. The ctDNA/cfDNA ratio is also affected by blood sampling and collection tubes. Indeed, the collection of serum stimulates a release of necrotic DNA from blood cells. This mechanism might account for the lower ctDNA/cfDNA ratio found in serum compared with plasma samples. Accordingly, current evidence suggests that the optimal specimen type for analysis of ctDNA in blood is plasma and not serum. Plasma collected in ethylenediamine tetraacetic acid tubes must be quickly separated from the blood cellular component within 6 hours to avoid the dilution effect of ex vivo cfDNA release by white blood cell lysis, which in turn decreases the ctDNA/cfDNA ratio. Cell-stabilizing tubes avoid white blood cell lysis, thus preserving the ctDNA/cfDNA ratio up to 96 hours after collection. There is consensus among studies that plasma must be isolated before freezing and storage, that DNA extraction from thawed plasma has no effect on ctDNA integrity, and that a single freeze-thaw cycle does not affect downstream ctDNA analysis. Therefore, current evidence suggests that processed plasma should be aliquoted into single-use fractions for future ctDNA extraction and analysis.^20^

### Controversy

Unsolved issues in the preanalytical handling of cfDNA have been discussed by a position paper of the American Society of Clinical Oncology and College of American Pathologists.^20^ Further methodological studies are needed to test the comparative effects of various blood draw variables, including the type of blood draw tube used, tube fill level, number of tube inversions, draw order if collected in one venipuncture with other blood draw tubes and patient-related factors that may contribute to the release of cfDNA (eg, diurnal or other biological influences, smoking, pregnancy, exercise, and numerous concomitant nonmalignant disorders such as inflammatory conditions, anemia, heart disease, metabolic syndrome, and autoimmune disorders). There are several different cfDNA purification methods, with different performance that may affect cell-free DNA yield and purity.^36^ Methodological studies should accurately measure differences in yield and quality of cfDNA and ctDNA by comparative testing of multiple methods and protocol parameters with the same plasma input.

## ARE STATE-OF-THE-ART CTDNA ASSAYS TECHNICAL VALIDATED AND FEASIBLE IN REAL-TIME?

### Polymerase chain reaction based: quantitative polymerase chain reaction; droplet digital polymerase chain reaction

Polymerase chain reaction (PCR)-based methods can study a single mutation at a known locus. Advantages of PCR-based methods are high sensitivity (sensitivity 80%-100%, detection limit of 10^−5^) and high specificity.^37^ A drawback is that they require a priori knowledge that the mutation specifically investigated by the assay is specific for the tumor.

An example of the application of droplet digital PCR (ddPCR) is the detection of the *MYD88* L265P mutation, which is a diagnostic biomarker of Waldenstrom macroglobulemia (WM), primary central nervous system lymphoma (PCNSL), and vitreoretinal DLBCL. In WM, Drandi et al^38^ showed that there was a good correlation of the detection of *MYD88* L265P mutation on plasma ctDNA and bone marrow findings.

In PCNSL, a ddPCR assay probing the hotspot *MYD88* L265P mutation has a sensitivity rate of 60% in plasma samples^39^ and could potentially be used on cerebrospinal fluid (CSF) samples.

Finally, in vitreoretinal DLBCL, ddPCR can detect the *MYD88* L265P mutation in vitreous fluid (another kind of liquid biopsy) with high sensitivity.^40^

Another example of the possible application of ddPCR is in cHL, where the detection of XPO1 hot spot by ddPCR may be used as biomarker for diagnosis and MRD.^41,42^

#### NGS: immunoglobulin sequencing, cancer personal profiling by deep sequencing, whole genome sequencing

NGS-based assays are able to detect immunoglobulin and T-cell receptor gene rearrangements, multiple classes of gene mutations, rearrangements, and copy number alterations. They are off-the-shelf approaches that do not require a prior knowledge of the mutation profile and clonality of the tumor and allow the identification of novel mutations occurring during clonal evolution of a tumor. Sensitivity of NGS assays incorporating chemistries or bioinformatics that suppress the error rate is comparable to that of PCR-based approaches, with a detection limit of 10^−5^.^10^ All these methods present technical challenges and their potential applications currently represent a field of intense investigation in lymphoma research.

Among these methods, immunoglobulin high-throughput sequencing (Ig-HTS) involves identification and tracking of the unique immunoglobulin sequence from the malignant B cells by using universal primers that target immunoglobulin genes. These methods have been studied in a variety of lymphoma subtypes, including DLBCL and MCL.^43^

Ig-HTS can track the clonal heterogeneity and clonal evolution in lymphomas comparing immunoglobulin somatic mutations at diagnosis and during the follow-up as demonstrated in FL and DLBCL, although when tumor IgH sequences are not available, IgH-HTS may be less performant.^28,44,45^

The CAncer Personalised Profiling by deep Sequencing (CAPP-seq) is a sensitive tool used to detect disease-specific mutations in ctDNA.^44,46^ This method utilizes a disease-specific selector (set of exonic and intronic targets chosen to cover regions of known recurrent mutations) to selectively sequence genomic regions of interest in a patient sample. CAPP-seq can in theory simultaneously test all important classes of mutations, including single nucleotide variants (SNVs), insertions/deletions (indels), copy number alterations and rearrangements.^47^ Given its high sensitivity and ability to reliably identify patient-specific target sequences, this NGS assay has been used in highly promising studies in lymphomas.^45,46,48^

Another approach to analyze ctDNA is an untargeted strategy represented by whole genome sequencing (WGS) which can convincingly detect somatic copy number aberrations. This method is less sensitive but does not require prior information on the tumor genome and allows identification of new mutations which may develop during treatment^49^ (Figure 1).

### Controversy

#### Deep or broad sequencing?

As mentioned before, 2 strategies have been developed to study ctDNA: the first is based on querying tumor specific mutations and uses assays characterized by high sensitivity and specificity but analyzing a limited number of known mutations (PCR-based approach), while an untargeted approach performs a more extensive study of the genome but with low sensitivity and requiring high amounts of ctDNA (NGS-based approach).

The limited sensitivity of the NGS methods used for the detection of ctDNA is a critical aspect for the use of this tool for the monitoring of MRD. To solve this problem, several bioinformatic pipelines have been developed to increase the sensitivity of mutation detection and decrease the sequencing error rate, in particular for monitoring residual disease. Kurtz et al^50^ recently described phased variant enrichment and detection sequencing (PhasED-seq) method, which is able to identify multiple somatic mutations in individual DNA fragments improving the sensitivity of ctDNA detection. Alternative bioinformatic approaches utilizing unique molecular identifiers in combination with bioinformatic error reduction using individual base error likelihoods as well as error likelihoods in base triplets have been proposed.^51^

In the context of MRD study where tumor fraction is low, deep sequencing methods with limited genomic targets have been applied. However, many patients with radiologically evident disease after treatment do not show detectable ctDNA. Deep-targeted NGS approaches are hindered by a fundamental barrier to detection sensitivity, namely the limited input material (the number of cfDNA fragments as measured by genomic equivalents). This may be particularly relevant in the settings of early cancer or MRD detection. In a typical plasma sample, cfDNA fragments are often in the range of only hundreds to several thousand genomic equivalents per milliliter. The limited number of genomic equivalents effectively places a ceiling on the depth of sequencing beyond which available distinct fragments are exhausted. The limited cfDNA input cannot be overcome through improvements of sample processing. Therefore, the only way of increasing cfDNA input would be maximizing blood collection from patients.

The sampling limitation can be effectively overcome by increasing the chance of detecting a ctDNA fragment through increased breadth of sequencing. Indeed, if the number of interrogated sites is wide as the genome, the chance of detecting a mutant ctDNA becomes dependent on the total number of somatic mutations within a typical tumor genome rather that the depth of sequencing (eg, if the cancer genome harbors 10,000 mutations, the anticipated sensitivity of this approach is 10^−4^). In DLBCL, the total number of somatic SNVs detected per case range from 1.000 to 50.000, with an average of 12.000 somatic mutations.^52^ Consistently, Zviran et al^36^ demonstrated in clinical cohorts of lung cancer, CRC, and melanoma that the use of WGS of cfDNA with a modest sequencing depth (ca. 30×) allowed ultrasensitive detection with a sensitivity of 10^−5^ demonstrating that breadth can overcome the depth of sequencing.

## WHAT ARE THE CLINICAL APPLICATIONS OF CTDNA ASSAYS IN LYMPHOMA?

### Can ctDNA be used for lymphoma diagnosis?

Although a mutation profile revealed by ctDNA can never replace the gold standard of histological tissue-based diagnosis, liquid biopsy represents a potential tool for lymphoma diagnosis in certain clinical situations, such as inaccessible tumor sites, deep tumor masses, and/or when the diagnosis is expected to result in limited treatment applications in unfit patients. A characteristic scenario is represented by PCNSL with surgically inaccessible mass. The gold standard for the diagnosis of PCNSL is stereotactic biopsy or, alternatively, if ocular or CSF involvement is evident, vitrectomy or CSF cytology may be sufficient. A less invasive technique could contribute to the diagnosis of PCNSL when biopsy of the brain lesion is not possible. A study of patients with CNS malignancies showed that 62% of patients had detectable genomic alterations in cfDNA collected from the cerebral spinal fluid.^53^ A second study showed that pretreatment ctDNA is detectable in 78% of plasma samples and 100% of CSF samples^54^; moreover, cases of isolated CNS relapse of DLBCL captured by plasma ctDNA have been reported.^45^ The *MYD88* L265P mutation is a characteristic of PCNSL and ddPCR assays probing the hotspot *MYD88* L265P mutation have a sensitivity rate of 60% in plasma samples^39^ and could potentially be used on CSF samples.

Another difficult diagnostic setting is represented by intravascular large B-cell lymphoma (IVLBCL), a rare lymphoma entity whose conventional diagnosis is based on the presence of lymphoma cells infiltrating blood vessels. In a small series of patients with IVLBCL among 9 patients with paired (tissue and plasma) samples available, allele frequency of mutations detected by NGS (*CD79B*, *MYD88*, *PIM1*, *PRDM1*, or *BTG2*) was higher in cfDNA from peripheral blood compared with genomic DNA from bone marrow or random skin biopsies.^55^ Futhermore, Shimada et al studied provided additional insights on the molecular pathogenesis of IVLBCL, identifying among others PD-L1, PDL2 and other genes in cfDNA.^56,57^ These data demonstrate that ctDNA analysis is complementary to clinical and pathological results and supports its potential usefulness for the diagnosis of lymphoma residing in poorly inaccessible anatomical sites.

#### Controversy

The diagnostic accuracy for PCNSL of detecting the *MYD88* L265P mutation in a consistent clinical and radiological context is still controversial, suggesting the need for well-designed, prospective studies based on standardized techniques before introducing cfDNA analysis in the diagnostic work up of PCNSL.

Further confounding the scenario is the well-documented notion that the *MYD88* L265P mutation occurs in other clonal diseases which are quite common in elderly patients, for example, monoclonal gammopathies of undetermined significance and monoclonal B-cell lymphocytosis. Such background conditions can raise the risk of a false positive claim of PCNSL in the case of casual co-occurrence of a brain mass in the same subject^58^ (Table 1).

**Table 1.** Liquid Biopsy in Lymphoma: Potential Clinical Applications and Controversies

Potential Clinical Applications	Controversies
Can ctDNA be used for diagnosis?	The same tumor mutation can occur also in other clonal diseases increasing the risk of false positives
Can genotyping of baseline ctDNA be used to guide treatment?	The mutation profile of the majority of tumors does not influence treatment decisions at the moment
Can ctDNA be used for MRD monitoring?	Several techniques are used at the moment but there is no consensus on the most sensitive approach

CtDNA = circulating tumor DNA; MRD = minimal residual disease.

### Can ctDNA be used to guide lymphoma treatment?

Several studies consistently showed that pretreatment ctDNA levels correlate with lymphoma tumor burden and its proxies, such as Ann Arbor stage, lactate dehydrogenate levels, International prognostic index (IPI), and total metabolic tumor volume.^28,29,59^ Consistently, high ctDNA load at baseline is a poor prognostic biomarker.

Genotyping of baseline ctDNA provides an average of genetic information within the tumor that may complement or replace information from tissue biopsies, which may be relevant if actionable mutations are used as predictive biomarkers for treatment tailoring.

A challenging question in the management of patients with lymphoma is how to monitor residual disease with high enough sensitivity, both during treatment and in post-treatment surveillance. ctDNA measurement allows for dynamic monitoring of residual disease, which complements and enhances conventional imaging (ie, 18 fluorodeoxyglucose positron emission tomography/computed tomography scan) in lymphoma restaging.^60^ Kurtz et al^29^ have shown in a large study that ctDNA was detectable by CAPP-Seq in 98% of DLBCL patients at baseline and pretreatment ctDNA levels highly correlated with disease burden. Moreover, they found an optimal threshold to predict outcome; a 2-log drop in ctDNA after 1 cycle (defined as early molecular response [EMR]) and a 2.5-log drop after 2 cycles (defined as major molecular response [MMR]) predicted Event-free survival in 2 validation sets of patients in front-line therapy.^29^

In this context, it has been proposed the continuous individualized risk index, a method based on the integration of 6 complementary risk predictors, including 3 established risk factors (IPI, cell of origin, and interim imaging) and 3 ctDNA risk factors (pretreatment ctDNA levels, EMR, and MMR) to dynamically determine the outcome of patients with DLBCL.^61^

In classical HL, a 2-log-fold reduction in ctDNA levels measured by CAPP-Seq after 2 cycles of doxorubicine, bleomycine, vinblastine, dacarbazine in patients with advanced stage disease was also strongly associated with PFS; conversely, a drop of less than 2-log in ctDNA after 2 treatment courses is associated with an eventual progression.^32^ Another study in 121 patients showed in a cohort consisting of early and advanced stage classical HL patients that MRD measured by ctDNA reduction can differentiate good and bad outcomes to treatment as early as 1 week after initiation of treatment.^51^ ctDNA is being investigated in lymphoma and in particular in DLBCL and HL as a tool that could allow early and accurate identification of chemorefractory patients who are candidates for treatment intensification, or the identification of good-risk patients, for treatment de-escalation.

On the other hand, the use of ctDNA for measuring remaining disease at an early interim timepoint may represent an early marker of treatment resistance in DLBCL patients treated with a chemo-free regimen. For example, ctDNA levels at day 28 after CAR-T cell infusion were shown to be prognostic in DLBCL.^34^ In another study, a significant increase in ctDNA at day 15 strongly associates with lack of response in lymphoma patients treated with panobinostat.^33^

Post-treatment surveillance has typically been performed by conventional scans (although routine imaging surveillance is no longer recommended), but several reports have highlighted the high sensitivity of liquid biopsies to detect disease recurrence before there is clinical evidence of relapse. An analysis of serial serum samples and concurrent CT scans during 5 years of follow-up in patients with DLBCL showed that resurgence of detectable ctDNA during follow-up strongly associates with subsequent clinical disease progression, with a median time of 3.5 months before CT scan positivity.^28^

#### Controversy

Baseline ctDNA load can replace current staging criteria only if it is documented to outperform modern imaging techniques that are conventionally used for tumor load estimation. Likely, ctDNA load will complement, but not substitute, baseline imaging, as it may capture biological features that are masked to imaging. Tumor aggressiveness is characterized by uncontrolled proliferation that is associated with high levels of apoptosis. As a consequence, aggressive tumors with rapid turnover proliferation and apoptosis are more prone to release high levels of apoptosis-derived ctDNA into the blood. In this scenario, ctDNA may vary considerably within the same disease stage of a given lymphoma type and herald prognostic information in addition to tumor volume.

Baseline lymphoma genotyping can be achieved by studying genomic DNA isolated from a tumor biopsy. Exceptions are cHL and WM, where ctDNA genotyping overcomes the technical hurdles imposed by the need of isolating the scattered tumor cells that the biopsy contains, and relapsed lymphomas, where often limited tumor material is available as it is often collected through core needle biopsies which can be insufficient for NGS assays. The more critical question is whether genotyping of lymphoma is clinically needed, as currently with only few exceptions (such as *MYD88* L265P and *CXCR4* mutations in WM, *EZH2* mutation in FL, *TP53* mutation in small lymphocytic lymphoma) mutation profile of the tumor does not affect treatment decisions (Table 1). This may change in the future, for example, if genomic subtyping of DLBCL will become more relevant for making treatment decisions.

MRD detection in cfDNA has several technical challenges. Therefore, harmonization of techniques (including bioinformatic analysis pipelines) and consensus on the most sensitive approach to be carried forward (ie, ultradeep targeted NGS of a dedicated gene panel versus whole genome sequencing) are urgently needed to ensure accurate and reproducible detection across centers (Table 1).

Despite positive results, the current literature on MRD detection in cfDNA is based on retrospective cohorts. Prospective studies are now needed to establish the feasibility of real-time ctDNA evaluation for diagnosis and follow-up as well as the most appropriate timing of collection. It will need to be determined how ctDNA integrates with established imaging for complementary monitoring of treatment response, and how to measure the utility of ctDNA as an earlier surrogate endpoint and in risk-adapted clinical trials.

## CONCLUSION

ctDNA is a promising and emerging lymphoma biomarker and represents a potential tool for diagnosis, prognosis, and monitoring of disease. The main critical aspect remains sensitivity and the lack of standardization of preclinical techniques, the need for technological standardization and harmonization, a consensus on the threshold nominating high ctDNA levels in pretreatment samples, and MRD negativity during/after treatment. At present, there are no published data on prospective studies based on a liquid biopsy-driven approach in lymphoma patients but some of them are ongoing for clinically validating ctDNA in lymphomas (NCT02661503, NCT04604067, NCT04866654, NCT04401774, NCT03758989) with the aim of studying ctDNA as a baseline biomarker to identify high-risk patients and also to define its role as a tool for disease response assessment. Further technological assessments and clinical studies will be required to validate this tool.

## SOURCES OF FUNDING

This work was supported by the grant: “Molecular bases of disease dissemination in lymphoid malignancies to optimize curative therapeutic strategies”, (5 x 1000 No. 21198); Associazione Italiana per la Ricerca sul Cancro Foundation Milan, Italy. SB received Deutsche Forschungsgemeinschaft (DFG) grants EN 179/13-1 and BO 5316/2-1, the HL MRD consortium, and the Frauke-Weiskam und Christel Ruranski-Stiftung.

## DISCLOSURES

SB is a shareholder founder and CEO of Liqomics, a liquid biopsy company. GG is in the advisory board of Abbvie, Astra-Zeneca, Beigene, Incyte, Janssen, Roch and speaker’s bureau Abbvie, Janssen. DR received honoraria from Janssen, AbbVie, and AstraZeneca, and research grants from Janssen, AbbVie, and AstraZeneca. All the other authors have no conflicts of interest to disclose.
